# Editorial: Peritoneal Metastasis of Gastric Cancer: From Basic Research to Clinical Application

**DOI:** 10.3389/fonc.2022.880497

**Published:** 2022-06-28

**Authors:** Heng Zhou, Ziming Gao, Anqi Sun, Haibo Huang, Xiaotian Zhang, Kai Li

**Affiliations:** ^1^ Department of Surgical Oncology, The First Hospital of China Medical University, Shenyang, China; ^2^ Department of Anesthesiology, The First Hospital of China Medical University, Shenyang, China; ^3^ Department of Gastrointestinal Oncology, Key Laboratory of Carcinogenesis and Translational Research (Ministry of Education/Beijing), Peking University Cancer Hospital and Institute, Beijing, China

**Keywords:** peritoneal metastasis, gastric cancer, editorial, basic research, clinical application

Gastric cancer (GC), with a 5-year survival rate of 20%-40%, ranks fifth for tumor incidence and fourth for cancer-related mortality globally, which is responsible for over one million new diagnosed cases in 2020 and an estimated 769,000 deaths (1). Practically, the poor prognosis and high mortality rate of GC are primarily due to metastasis. Notably, previous studies reported that almost 40% of patients with stage II-III gastric cancer were diagnosed with peritoneal metastases (PM) intraoperatively, and over 50% of GC patients show PM after radical resection (2). Particularly, GC patients with PM achieve exceptionally poor survival outcomes with a median overall survival of only 2-9 months (3). Biologically, PM is an extremely complicated process involving detachment from the primary tumor, seeding and survival in the cavum abdominis, adhesion to the peritoneum, invasion through the basement membrane to sub-peritoneal tissue, and proliferation with blood vascular neogenesis. Meanwhile, epithelial-mesenchymal transition (EMT) is essential for epithelial-derived malignant tumor cells to acquire the ability of migration and invasion, and cellular stemness can effectively suppress anoikis of free GC cells in the abdominal cavity and promote colonization in the peritoneal milky spots (4–6). Clinically, PM cannot be detected by conventional imaging techniques, intraperitoneal cytology, or diagnostic laparoscopy due to unformed peritoneal cancer nodules or minimal exfoliated cancer cells. Therefore, the mechanism, diagnosis, and treatment of PM remain daunting challenges in oncology. To effectively avert PM-related deaths, it is necessary to pay more attention to the fundamental and clinical research on PM.

The Research Topic of Peritoneal Metastasis of Gastric Cancer makes a deep study of the molecular mechanisms and clinical application of PM, which provides readers with a new understanding of PM. Based on the classical “seed and soil” theory, metastasis is linked to the characteristics of the malignant tumor, that is the ability of the “seed”. Malignant tumor cells are highly invasive and can directly spread to adjacent sites, or spread to distal sites through circulatory systems such as lymphatic vessels and blood vessels, or are directly shed and implanted into other sites. Meanwhile, metastasis is related to the tumor microenvironment (TME), that is the “soil”.

To further explore the mechanisms of PM, Gao et al. extensively describe the role of exosomes in gastric cancer malignity and pre-metastatic niche formation. Specifically, exosomes participate in the promotion of gastric cancer growth, the induction of drug resistance, and the facilitation of metastasis. Furthermore, exosomes promote PMN formation *via* immunosuppression, stroma remodeling, inducing PM cells mesothelial-mesenchymal transition, angiogenesis and organotropism. Additionally, exosomes have great potential as biomarkers and drug delivery carriers for cancer detection, prognosis, prevention, and treatment.

Proverbially, the immune microenvironment plays a crucial role in tumor biology. Jiang et al. describe the immune cell landscape based on the ESTIMATE and CIBERSORT algorithms to cluster GC into 3 subtypes. Moreover, they establish and validate an immune cell infiltration scoring system to predict the survival and chemotherapy responsiveness of GC patients based on public databases and their own.

Detection of PM counts on advanced imaging tools. However, it is not sensitive enough to perceive the peritoneal cancer nodules <5mm (7). Recently, deep learning and artificial intelligence (AI) have shown extraordinary performance in image processing which overcome the limitation of handcrafted imaging and manual labeling. Chen et al. perform dual-energy computed tomography-based radiomics to predict PM in 239 recruited GC (non-PM = 174, PM = 65) patients. Boruta and Spearman correlation analyses were utilized for feature selection. Subsequently, a random forest was used to tune the optimal radiomics model. Finally, the model constructed by the features extracted from standardized iodine-uptake images demonstrated significantly better performance to predict PM. As expected, it provided a noninvasive, easy-to-use, and representative tool to preoperatively predict PM for GC.

Advanced gastric cancer (AGC) is characterized by a poor prognosis and high peritoneal recurrence. What is an ideal therapy to prevent peritoneal recurrence after AGC radical resection? Generally, the therapy, which can effectively kill the remaining free cancer cells and subclinical lesions in the abdominal cavity intraoperatively or postoperatively and maximize the radical treatment at both macro and micro levels, is considered to be effective. Xu et al. perform a multi-center, randomized, open-label, controlled clinical study to investigate the efficacy of intraoperative sustained-release fluorouracil implants after radical resection combined with postoperative adjuvant chemotherapy for cTNM stage III gastric cancer. Totally, 122 patients were enrolled and randomized into the intraperitoneal chemotherapy group and control group. Compared with the control group, the peritoneal recurrence was significantly decreased in the intraperitoneal chemotherapy group, which could effectively prolong progression-free survival.

In conclusion, discovering how to break through the difficulties and find the feasibility of preventing and treating PM is of great present urgency. Therefore, we have the obligation to cope with the challenge. This Research Topic collects vital reviews and original research, which circumstantiates the PM systematically and provides an objective basis for the diagnosis and treatment of peritoneal metastasis of gastric cancer.

## Author Contributions

All authors contributed to the article and approved the submitted version.

## Conflict of Interest

All authors declare that the research was conducted in the absence of any commercial or financial relationships that could be construed as a potential conflict of interest.

## Publisher’s Note

All claims expressed in this article are solely those of the authors and do not necessarily represent those of their affiliated organizations, or those of the publisher, the editors and the reviewers. Any product that may be evaluated in this article, or claim that may be made by its manufacturer, is not guaranteed or endorsed by the publisher.
